# Effects of Supplements Differing in Fatty Acid Profile to Late Gestational Beef Cows on Steer Progeny Finishing Phase Growth Performance, Carcass Characteristics, and mRNA Expression of Myogenic and Adipogenic Genes

**DOI:** 10.3390/ani11071904

**Published:** 2021-06-26

**Authors:** Taoqi Shao, Joshua C. McCann, Daniel W. Shike

**Affiliations:** Department of Animal Sciences, University of Illinois, Urbana, IL 61801, USA; taoqis2@illinois.edu (T.S.); jcmccan2@illinois.edu (J.C.M.)

**Keywords:** beef cattle, carcass characteristics, fatty acids, finishing performance, maternal supplementation, mRNA expression

## Abstract

**Simple Summary:**

Maternal nutrition during late gestation affects fetal muscle and adipose tissue development, leading to persistent impacts on offspring postnatal growth and production performance. Fatty acids, especially essential fatty acids, play important roles in regulating protein and lipid metabolism. Therefore, the objective of the current study was to investigate the effects of maternal fatty acid supplementation during late gestation on beef cattle progeny finishing phase growth performance, carcass characteristics, and mRNA expression of genes involved in muscle and adipose tissue development. This study demonstrated that maternal supplementation with polyunsaturated fatty acids during late gestation increased the offspring’s feed ratio during the finishing phase. However, it had limited effects on finishing phase body weights, carcass characteristics, or relative mRNA expression in *longissimus* muscle and subcutaneous adipose tissue. Therefore, operations under similar systems could expect limited effects of maternal fatty acid supplementation on the growth and production performance of the finishing steers.

**Abstract:**

The objective was to investigate the effects of feeding late gestational beef cows supplements differing in fatty acid profile on steer progeny finishing phase growth performance, carcass characteristics, and relative mRNA expression of myogenic and adipogenic genes. Seventy Angus-cross steers (initial body weight [BW] 273 ± 34 kg) born from dams supplemented with either 155 g DM/d EnerGII (CON, rich in palmitic and oleic acids) or 80 g DM/d Strata + 80 g DM/d Prequel (PUFA, rich in linoleic acid, eicosapentaenoic acid, and docosahexaenoic acid) for the last 77 ± 6 d prepartum were used. *Longissimus* muscle and subcutaneous adipose biopsies were collected to evaluate relative mRNA expression of genes related to myogenesis and adipogenesis. Steers were slaughtered at 423 ± 6 d of age. No treatment × time interaction or treatment effect (*p* ≥ 0.21) was detected for steer finishing phase BW, while steers from PUFA supplemented dams tended (*p* = 0.06) to have a greater gain to feed ratio (G:F). Neither carcass characteristics nor relative mRNA expression was different (*p* ≥ 0.11). In conclusion, late gestation PUFA supplementation tended to increase steer progeny finishing phase G:F, but had no effects on finishing phase BW, carcass characteristics, or relative mRNA expression during the finishing phase.

## 1. Introduction

Maternal nutrition can have fetal programming effects. During late gestation, restriction of maternal nutrients reduces the diameter of muscle fibers and density of satellite cells, which negatively impact postnatal muscle growth [1,2]. Marbling is crucial for meat palatability and is correlated with the number and size of intramuscular adipocytes [3]. The neonatal period is a major stage for developing intramuscular adipocytes [4]. Manipulating the maternal supplementation of nutrients during late gestation is expected to modify intramuscular adipogenesis [5]. Therefore, it is critical to provide adequate nutrients for optimal early development of muscle and adipose tissue and subsequent postnatal health and growth performance.

Recent fetal programming studies have focused on specific maternal nutrients, for example, essential fatty acids [6,7,8,9,10] and one-carbon metabolites [11,12,13] on offspring health and growth performance. Maternal supplementation of rumen-protected fatty acids during late gestation can modify the essential fatty acid status of the offspring [14]. In addition, essential fatty acids are important for regulating developing muscle and adipose tissue [15,16,17,18,19]. For instance, n-3 fatty acids are important for the function development of bovine *Longissimus* muscle [17]. On the other hand, the master regulator for adipogenesis, Peroxisome proliferator-activated receptor gamma (*PPARG*), is stimulated by n-6 fatty acids [18]. Therefore, supplementing fatty acids during late gestation could manipulate the health and growth performance of the offspring.

Recent studies in ruminants investigated the finishing performance of progeny from dams that received polyunsaturated fatty acids. The supplementing of late gestation beef cows with polyunsaturated fatty acids [6,8] improved finishing phase growth performance and carcass quality. In addition, lambs from dams supplemented eicosapentaenoic acid (EPA) and docosahexaenoic acid (DHA) had greater finished BW [7]. However, work investigating maternal supplementation of fatty acids in fall-calving grazing systems is lacking. Furthermore, limited data are available for fetal programming effects of maternal fatty acid supplementation on mRNA expression of the myogenic and adipogenic genes during the post-weaning stage. Therefore, the objective of this study was to evaluate the effects of supplements differing in fatty acid profile to late gestation beef cows on steer progeny finishing phase growth performance, carcass characteristics, and relative mRNA expression of genes associated with myogenesis and adipogenesis. It was hypothesized that steers from dams supplemented with polyunsaturated fatty acids during late gestation would have better finishing phase growth performance and improved carcass merit.

## 2. Materials and Methods

Experimental animals were managed according to the guidelines recommended in the Guide for the Care and Use of Agricultural Animal in Agricultural Research and Teaching (Federation of Animal Science Societies, 2010). In addition, all experimental procedures were approved by the Institutional Animal Care and Use Committee of the University of Illinois (IACUC #18109).

### 2.1. Experiment Design, Animals, and Management

This study was conducted at the Beef Cattle and Sheep Field Laboratory in Urbana, IL, USA. Seventy Angus-cross steers were blocked into heavy and light groups based on weaning BW and assigned into 4 pens with 17 or 18 steers per pen. Within each block, previous dam treatments (*n* = 2), dam grazing groups (*n* = 8), and sire (*n* = 7) had similar representations in each pen. The management of the steers and their dams during the preweaning period was published by Shao et al. [20]. In brief, steers were born from fall-calving beef cows supplemented 0.77 kg dry matter (DM)/d soybean hulls mixed with either 155 g DM/d EnerGII (CON) or 80 g DM/d Strata + 80 g DM/d Prequel (PUFA) for the last 77 ± 6 d of gestation. Supplementation diets were designed to be isocaloric and isonitrogenous, with only differences in fatty acid profile. CON was rich in saturated and monounsaturated fatty acids, while PUFA was rich in polyunsaturated fatty acids (Table 1). Virtus Nutrition LLC, Corcoran, CA, provided calcium salts of fatty acids (Strata, Prequel, and EnerGII). Cows were grazing on 8 predominately endophyte-infected tall fescue pastures (*n* = 4 pastures/treatment) and supplemented 3 times weekly during the supplementation period. Forage during the supplementation period had 1.5% total fatty acid, of which it had 20.4% C16:0, 20.1% C18:2n-6, and 40.1% C18:3n-3. Soybean hulls had 1.0% total fatty acid, comprising 17.2% C16:0, 14.1% C18:1c9, and 38.6% C18:2n-6. Once a week, cows that had calved and their calves were removed from treatment grazing groups to a common pasture for commingled management from that point forward. The average supplementation length was 81 ± 7 d.

Housing conditions for the steers were previously reported by Segers et al. [21]. Pen size for the current study was 14.64 m × 4.88 m in dimension. Day 0 of the experiment is defined as the first-day steer progeny received their finishing diet. Upon arrival (day −42), the steers were given access to a receiving ration in concrete bunks for 1 week. After the first week (day −34), steers were provided with step-up transition diets for 33 days (Table 2). During the last two weeks of the transition phase, steers were adapted to GrowSafe feed bunks (GrowSafe System Ltd., Airdrie, AB, Canada) for 190-d finishing phase individual daily feed intake measurement. After that, the full BW of the steers was measured for two consecutive days for the initial (days 0 and 1) and the end (days 189 and 190) of the finishing phase. Besides the initial and final two-day weighing, BW of the steers was also measured on days 28, 56, 83, 111, 139, and 167 to monitor growth performance. Steers were implanted on day 0 with Component TE-IS (16 mg estradiol and 80 mg trenbolone acetate; Elanco Animal Health, Greenfield, IN, USA). On day 83, they were implanted with Component TE-S (120 mg trenbolone acetate, 24 mg estradiol USP, and 29 mg tylosin tartrate; Elanco Animal Health, Greenfield, IN, USA). Steers were fed 300 mg/steer/day of Optaflexx 45 (100 g ractopamine hydrochloride/kg, Elanco Animal Health, Greenfield, IN, USA) for the last 30 d on feed. Slaughter date was selected by visually appraising to target an average of 1.7 cm 12th rib fat thickness. On day 190, steers (average 423 ± 6 days of age) were shipped to a commercial abattoir (Tyson Fresh Meat, Joslin, IL, USA) and harvested humanely. Hot carcass weight (HCW) was measured upon slaughter, and after a 24 h chill, the USDA camera system assessed carcass characteristics. The USDA equation calculates yield grade.

### 2.2. Sampling and Analytical Procedures

Feed samples for each ingredient were collected from common diets fed to steers during receiving, transition, and finishing phases. Feed sampling was conducted every 28 days throughout the finishing phase. Individual ingredients were composited, dried, and ground at the end of the finishing phase. All feed samples were analyzed for NDF and ADF (using Ankom Technology method 5 and 6, respectively; Ankom200 Fiber Analyzer, Ankom Technology, Macedon, NY, USA), fat (using Ankom Technology method 2; Ankom XT10 Fat Analyzer, Ankom Technology), CP (Leco TruMac, LECO Corporation, St. Joseph, MI, USA), and ash (600 °C for 2 h; Thermolyne muffle oven Model F30420C, Thermo Scientific, Waltham, MA, USA).

Three weeks before slaughter (day 168), biopsies (*n* = 22) were collected from *longissimus* muscle and tail-head subcutaneous adipose tissue of the previously selected steers [20]. This was used to analyze relative mRNA expression for genes related to myogenesis and adipogenesis. Three steers were biopsied from each cow grazing group during previous collections, while one steer died during the finishing phase because of tail surgery, and one steer was failed to be collected during biopsy sampling. These two non-biopsied steers came from different treatments. The average age of the biopsied steers was 401 ± 4 d. The biopsy procedures for muscle and adipose tissue were reported previously by Shao et al. [20]. Biopsy samples were snap-frozen in liquid nitrogen and stored at −80 °C for further processing and analysis.

Extraction of RNA from the biopsy sample, the primers used for qPCR, and the analysis of relative mRNA expression were previously described by Shao et al. [20]. There were no treatment effects on the abundance of any of the housekeeping genes. Primer qPCR performance for the target genes is presented in Supplementary Tables S1 and S2.

### 2.3. Statistical Analysis

Cow group was the experimental unit. Data were analyzed with the MIXED procedure of SAS (version 9.4; SAS Institute Inc., Cary, NC, USA). Cow group nested within treatment was included as a random effect for all response variables [23]. Outliers were checked by using the PROC REG procedure of SAS, removing data with a studentized t > 3.0 before analysis. The repeated measure was used for the analysis of steer BW during the finishing phase. Based on the Akaike information criterion, ANTE(1) was used as the covariate structure. The model included treatment, time, finishing pen nested within BW block, and the interaction between treatment and time as fixed effects, and expected progeny differences (EPD) of yearling weight as a covariate. The models used for the analysis of average daily gain (ADG), dry matter intake (DMI), and gain to feed ratio (G:F) included treatment as a fixed effect, while the EPD of ADG and finishing pen nested within BW block as covariates. The model used for carcass characteristics included treatment as a fixed effect and EPDs of the specific parameter and finishing pen nested within BW block as covariates. Data concerning relative mRNA expression that were not normally distributed were transformed as described by Shao et al. [20]. The model used to analyze relative mRNA expression included treatment as a fixed effect and sire as a random effect. Treatment effects were considered significant at *p* ≤ 0.05 and tendencies at 0.05 < *p* ≤ 0.10.

## 3. Results

### 3.1. Steer Growth Performance during Finishing Phase

Finishing phase BW is presented in Table 3. No treatment × time interaction (*p* = 0.27) or treatment effect (*p* = 0.21) for steer progeny finishing phase BW was observed. There were no differences in ADG or DMI, while steers from PUFA supplemented dams tended (*p* = 0.06; Table 4) to have greater G:F than CON steer.

### 3.2. Carcass Characteristics

Steer progeny carcass characteristics, including HCW, dressing percentage, yield grade, LM area, marbling score, 12th rib fat thickness, or KPH, were not different (*p* ≥ 0.22; Table 5) between treatments. 

### 3.3. Relative mRNA Expression

#### 3.3.1. *Longissimus* Muscle

There were no differences (*p* ≥ 0.11; Table 6) in relative mRNA expression of the selected myogenic or adipogenic genes at the finishing phase between CON and PUFA.

#### 3.3.2. Subcutaneous Adipose Tissue

There were no differences (*p* ≥ 0.11; Table 7) in relative mRNA expression of the selected genes at the finishing phase between CON and PUFA.

## 4. Discussion

As previously described [20], cows were rotationally grazing on tall fescue pastures with similar forage availability during the supplementation period. After that, grazing groups were commingled, and calves were managed the same from comingling through the finishing phase. Hence, any differences in animal responses should be attributed to late gestation supplementation, which was either rich in saturated and monounsaturated fatty acids or polyunsaturated fatty acids. Pre-weaning data have been published by Shao et al. [20]. Briefly, cow BW or body condition score was not different from the beginning of the supplementation to weaning. However, steer progeny from CON supplemented dams had greater weaning BW and tended to have greater ADG during the pre-weaning period than PUFA.

Based on previous studies [6,7], the current study hypothesized that steers from PUFA supplemented dams would have better finishing phase growth performance as well as improved carcass merit. Martin et al. [7] reported that lambs born from dams supplemented with Ca salts of EPA and DHA (PFA) during the last 50 d of gestation had greater BW at the end of the finishing phase compared to lambs from dams that received Ca salts of palmitic fatty acid distillate (C). However, the difference was minimal (52.2 kg vs. 53.6 kg for C and PFA, respectively). Recent studies [6,8] have investigated late gestation fatty acid supplementation during the winter to spring-calving beef cows with grass-alfalfa hay basal diets. Calves from dams fed 190 g/cow daily of Ca salts of EPA, DHA, and linoleic acid from d 195 of gestation to calving had greater growing phase ADG and tended to have greater finishing phase ADG, as well as greater final finishing BW [6]. Under the same production system as Marques et al. [6], Brandao et al. [8] reported that steers from dams supplemented with Ca salts of soybean oil during late gestation had greater ADG and final BW during the finishing phase. The authors speculated it could be partially attributed to less bovine respiratory disease (BRD) treatment needed. In the current study, steers from PUFA supplemented dams tended to have greater G:F during the finishing phase, but there were no differences in DMI or ADG. Animal studies [24,25,26] indicate that maternal nutrition impacts the appetite of the offspring during postnatal life. Martin et al. [7] reported no difference in DMI of the lambs during the finishing phase. Previous studies on beef cattle [6,8] did not report finishing phase intake data, while Santos et al. [27] reported that dairy calves born from saturated fatty acids-supplemented dams had greater DMI for the first 60 d of life and led to greater ADG. In the current study, BW of the steers from PUFA supplemented dams was 15 kg lighter than CON at the start of the finishing phase, which was due to CON had greater steer weaning BW [20]. However, there was no treatment by time interaction or treatment effect observed for finishing BW in the current study. Greater G:F’s tendency could be attributed to steers from PUFA supplemented dams narrowing the numerical differences in BW during the finishing phase.

However, offspring finishing growth performance in this experiment differs from recent studies on spring-calving beef cows supplemented during the winter [6,8] and sheep fed mixed ration [7]. Different operating systems may have contributed to different animal responses. Marques et al. [6] and Brandao et al. [8] had a spring-calving system with grass-alfalfa hay-based diet for cows. On the other hand, Martin et al. [7] had a limit-fed feeding system for ewes in pens. Differently, fall-calving beef cows were grazing on endophyte-infected tall-fescue pastures from July to October, which should have led to greater environment temperature and fescue toxicity. Heat stress during late gestation negatively affected growth performance on dairy calves from birth to 12 months of age [28]. In addition, heat stress [29] and fescue toxicity [30] can reduce placental blood flow and decrease nutrient supply, including fatty acid supply, to the fetus during critical developmental periods. Therefore, different environments and basal diets may have played an important role in differing animal responses. Supplementation profile and amount could also contribute to the limited effects on finishing phase growth performance in the current study. Future investigation is needed to validate the observations and test different scenarios.

Improved progeny carcass characteristics, including greater HCW and LM area, were reported when their dams had late gestation supplementation of polyunsaturated fatty acids [6,8]. In addition, Marques et al. [6] reported a greater marbling score of the calves born from dams supplemented with polyunsaturated fatty acids. However, the current study and Martin et al. [7] did not observe any differences in carcass characteristics. Late gestation maternal nutrient deficiency reduces muscle fiber size [31,32]. In addition, late gestation is critical for developing muscle satellite cells and intramuscular adipocytes [2,33]. The formation of intramuscular adipocytes during late gestation to early weaning stage determines the potential of marbling formation during later stages [33]. However, the current study indicates the limited effects of maternal supplementation of polyunsaturated fatty acids on postmortem carcass traits in a fall-calving production system.

Finishing phase relative mRNA expression of the genes associated with muscle and adipose tissue development supports the lack of difference in finishing phase performance and carcass characteristics. There were no differences detected in relative mRNA expression of any myogenic genes (*MYOG*, *MYOD1*, *PAX7*, *MYG5*, *MYH1*, *MYH2*, *MYH7*, and *MEF2C*) or adipogenic genes (*AGPAT1*, *PPARGC1A*, *PPARG*, *ZFP423*, *CEBPA*, *CEBPB*, and *FABP4*) in *Longissimus* muscle prior to slaughter at the end of the finishing phase. The tendency of steers from CON supplemented dams with greater relative mRNA expression of *Longissimus* muscle *MYF5* and *MYOG* during pre-weaning period [20] did not persist or transfer into differences in finishing phase growth performance. Similarly, greater relative mRNA expression of *CEBPB* at weaning in *Longissimus* muscle of steers from PUFA supplemented dams [20] did not persist or transfer into any differences in carcass characteristics. Consistent with the current study, Brandao et al. [8] reported that maternal supplementation of protected soybean oil during late gestation had no effects on mRNA expression of *Longissimus* muscle genes during the finishing phase. Both Brandao et al. [8] and the current study showed no effect of maternal fatty acid during late gestation on mRNA expression of *FABP4*, *MYOD*, *MYOG*, or *PPARG* in the *Longissimus* muscle of offspring during the finishing phase. Therefore, relative mRNA expression of *Longissimus* muscle genes may only be affected by maternal supplements differing fatty acids at early age of the offspring [8,20] but have limited effects later in the finishing phase prior to slaughter. In subcutaneous adipose tissue, maternal supplementation did not affect the mRNA expression of genes associated with adipogenesis or lipid metabolism. In sheep [15], there was a dam fat supplementation × lamb fat supplementation interaction detected for elongation of very long chain fatty acid 2 (*ELOVL2*) and hormone-sensitive lipase (*HSL*) in finishing lamb subcutaneous adipose tissue, but no dam fat supplementation effects were detected for adipogenic genes except one receptor gene glucose-dependent insulinotropic polypeptide (*GIP*). However, greater expression of *GIP* did not transfer to differences in plasma metabolites in lambs [15]. The current study did not evaluate *ELOVL2*, *HSL*, or *GIP*.

In contrast, both Coleman et al. [15] and the current study showed no effects of maternal fatty acid on mRNA expression of *FABP4*, *FASN*, *PPARG*, or *SCD* in subcutaneous adipose tissue of offspring during the finishing phase. Greater relative mRNA expression of *ZFP423* in steer subcutaneous adipose tissue from CON during the pre-weaning period [20] did not persist or transfer into any differences in finishing phase growth performance or postmortem carcass characteristics. Therefore, maternal supplements differing in fatty acid profile could have limited impacts on subcutaneous adipose tissue development during the finishing phase.

## 5. Conclusions

Supplementing PUFA to late gestational cows tended to increase steer progeny G:F during the finishing phase. However, there were no impacts on finishing phase BW, DMI, or ADG. Carcass characteristics were not affected by maternal late gestation fatty acid supplementation. No differences were observed for the relative mRNA expression of genes associated with myogenesis or adipogenesis during the finishing phase. Since this study was conducted under a fall-calving grazing system, no studies with similar production systems have been reported. Future investigations are needed to validate the observations and test different scenarios.

## Figures and Tables

**Table 1:** Nutritional profile and intake of the supplements that contained soybean hulls with 155. g DM/d EnerGII (CON) or 80 g DM/d Strata + 80 g DM/d Prequel (PUFA) for the last 77 ± 6 d of gestation.

Item ^1^	CON	PUFA
Ingredient, kg/cow/d		
Soybean hull	0.77	0.77
EnerGII	0.155	0
Strata	0	0.08
Prequel	0	0.08
Macronutrient intake, kg/cow/d		
Dry matter	0.93	0.93
Crude protein	0.07	0.07
Ether extract	0.16	0.15
Fatty acid intake, g/d		
C16:0	57.86	14.43
C18:0	5.74	6.97
C18:1c9	44.69	24.41
C18:2n-6	13.60	33.24
C18:3n-3	1.32	2.24
C20:5n-3	0	6.41
C22:6n-3	0	4.12

^1^ EnerGII, Prequel, and Strata were from Virtus Nutrition LLC, Corcoran, CA, USA.

**Table 2:** Diet and nutrient composition during receiving, adaptation, and finishing phase ^1.^

Item	Receiving	Transition 1	Transition 2	Finishing
Ingredient, % DM inclusion				
High moisture corn	10	25	40	50
Modified distillers grains	20	20	20	20
Corn silage	20	45	30	20
Supplement **^2^**	10	10	10	10
Grass hay	40	0	0	0
Chemical analysis, % DM				
Dry matter	64.0	51.1	56.8	58.0
Crude protein	13.9	13.1	13.2	13.2
Neutral detergent fiber	37.8	24.9	21.0	17.9
Acid detergent fiber	19.8	11.6	9.2	7.5
Ether extract	4.5	5.3	5.6	5.7
NE_m_^3^, Mcal/kg	1.64	1.89	2.00	2.07
NE_g_^3^, Mcal/kg	1.04	1.26	1.35	1.41

^1^ Receiving: d −42 to −35, Transition 1: d −34 to −28, Transition 2: d −27 to −1, Finishing: d 0 to 190; ^2^ Supplement contained 73.4% ground corn, 17.8% limestone, 6.7% urea, 1.0% trace mineral premix, 0.17% Rumensin 90, 0.11% Tylan 40, and 0.84% fat. Trace mineral premix contained 8.5% Ca, 5% Mg, 7.6% K, 6.7% Cl, 10% S, 0.5% Cu, 2% Fe, 3% Mn, 3% Zn, 278 mg/kg Co, 250 mg/kg I, 150 mg/kg Se, 2205 KIU/kg Vit A, 662.5 KIU/kg Vit D, 22,047.5 IU/kg Vit E. Tylan 40 (Elanco Animal Health, Greenfield, IN, USA);^3^ Net energy for maintenance (NE_m_) and gain (NE_g_) values are calculated according to NASEM, 2016 [22].

**Table 3:** Effects of supplementing cows with either 155 g DM/d EnerGII (CON) or 80 g DM/d Strata + 80 g DM/d Prequel (PUFA) for the last 77 ± 6 d of gestation on steer progeny finishing phase body weight.

Body Weight, kg	CON	PUFA	SEM	*p*-Value		
				Trt	Time	Trt × Time
Initial ^1^	280	265	3.3	0.21	<0.01	0.27
d 28	354	344	3.4			
d 56	401	390	3.6			
d 83	456	448	3.5			
d 111	503	497	3.9			
d 139	552	546	4.1			
d 167	596	590	4.6			
d 190	631	628	4.5			

^1^ The initial day of the finishing phase was considered d 0 of the experiment, and steers averaged 233 ± 6 days of age; SEM = standard error of the mean.

**Table 4:** Effects of supplementing cows with either 155 g DM/d EnerGII (CON) or 80 g DM/d Strata + 80 g DM/d Prequel (PUFA) for the last 77 ± 6 d of gestation on steer progeny finishing performance.

Item ^1^	CON	PUFA	SEM	*p*-Value
ADG, kg/d	1.86	1.92	0.035	0.13
DMI, kg/d	10.8	10.9	0.19	0.71
G:F	0.171	0.178	0.0036	0.06

^1^ ADG = average daily gain; DMI = dry matter intake; G:F = gain to feed ratio; SEM = standard error of the mean.

**Table 5:** Effects of supplementing cows with either 155 g DM/d EnerGII (CON) or 80 g DM/d Strata + 80 g DM/d Prequel (PUFA) for the last 77 ± 6 d of gestation on steer progeny carcass characteristics.

Items ^1^	CON	PUFA	SEM	*p*-Value
HCW, kg	385	386	5.5	0.86
Dressing percentage, %	60.8	61.2	0.37	0.29
12th rib fat thickness, cm	1.87	1.72	0.105	0.22
LM area, cm^2^	87.4	86.2	1.43	0.40
KPH, %	2.07	2.05	0.045	0.77
Yield grade	3.80	3.63	0.148	0.29
Marbling score ^2^	510	525	19.8	0.47

^1^ HCW = hot carcass weight; LM area = *longissimus* muscle area; KPH = kidney pelvic heart fat; SEM = standard error of the mean; ^2^ 400 = Choice USDA Quality Grade, 500 = Average Choice USDA Quality Grade, 700 = Prime USDA Quality Grade.

**Table 6:** Relative mRNA expression of genes regulating myogenesis and adipogenesis in *Longissimus* muscle of the steer progeny born from cows supplemented with either 155 g DM/d EnerGII (CON) or 80 g DM/d Strata + 80 g DM/d Prequel (PUFA) for the last 77 ± 6 d of gestation ^1.^

Genes ^2^	CON	PUFA	SEM	*p*-Value
*MYOG*	1.152	1.041	0.1363	0.45
*MYOD1*	1.183	1.073	0.1585	0.47
*PAX7*	1.070	0.956	0.1090	0.34
*MYF5*	1.413	1.275	0.1613	0.43
*MYH1*	1.264	1.218	0.1232	0.72
*MYH2*	1.184	1.022	0.1880	0.42
*MYH7*	1.170	1.129	0.1425	0.78
*MEF2C*	1.245	1.164	0.0845	0.38
*AGPAT1*	1.058	1.238	0.0975	0.11
*PPARGC1A*	0.353	0.725	-	0.32
*PPARG*	0.472	0.496	-	0.90
*ZFP423*	1.100	1.053	0.0493	0.38
*CEBPA*	0.352	0.436	-	0.67
*CEBPB*	0.293	0.318	0.0272	0.38
*FABP4*	0.298	0.249	-	0.79

^1^ Means are back-transformed if a transformation was conducted, SEM is calculated if data were normally distributed; ^2^ *MYOG* Myogenin, *MYOD1* Myogenic differentiation 1, *PAX7* Paired box protein 7, *MYF5* Myogenic factor 5, *MYH1* Myosin heavy chain 1, *MYH2* Myosin heavy chain 2, *MYH7* Myosin heavy chain 7, *MEF2C* Myocyte enhancer factor 2C, *AGPAT1* Acyl-glycerol phosphate acyltransferase 1, *PPARGC1A* PPARG coactivator 1 alpha, *PPARG* Peroxisome proliferator-activated receptor gamma, *ZFP423* Zinc finger protein 423, *CEBPA* CCAAT enhancer binding protein alpha, *CEBPB* CCAAT enhancer binding protein beta, *FABP4* Fatty acid binding protein 4.

**Table 7:** Relative mRNA expression of genes regulating adipogenesis in subcutaneous adipose of the steer progeny born from cows supplemented either 155 g DM/d EnerGII (CON) or 80 g DM/d Strata + 80 g DM/d Prequel (PUFA) for the last 77 ± 6 d of gestation ^1.^

Genes ^2^	CON	PUFA	SEM	*p*-Value
*FASN*	0.962	1.022	0.0871	0.52
*SREBP1*	1.090	1.309	0.1292	0.14
*PPARG*	1.070	1.095	0.0927	0.80
*ADFP*	1.081	1.079	0.1364	0.99
*SCD*	0.934	1.133	0.1059	0.11
*FABP4*	1.285	1.359	0.1300	0.59
*PPARGC1A*	1.009	1.043	0.1374	0.82
*ZFP423*	1.044	1.022	0.0547	0.70
*ACACA*	1.002	1.124	0.1416	0.42
*CEBPA*	1.085	1.195	-	0.49
*CEBPB*	0.875	0.937	-	0.54

^1^ Means are back-transformed if a transformation was conducted, SEM is calculated if data were normally distributed; ^2^ *FASN* Fatty acid synthase, *SREBP1* Sterol regulatory element-binding transcription factor 1, *ADFP* Adipose differentiation-related protein, *SCD* Stearoyl-CoA desaturase, *ACACA* Acetyl-CoA carboxylase alpha.

## Data Availability

The datasets used and/or analyzed are available from the corresponding author upon reasonable request.

## Supplementary Materials

The following are available online at https://www.mdpi.com/article/10.3390/ani11071904/s1, Table S1: qPCR performance of the genes analyzed in *Longissimus* muscle; Table S2: qPCR performance of the genes analyzed in adipose tissue
