# Supplementary material for: Effects of Supplements Differing in Fatty Acid Profile to Late Gestational Beef Cows on Steer Progeny Finishing Phase Growth Performance, Carcass Characteristics, and mRNA Expression of Myogenic and Adipogenic Genes
Source: Animals (Basel). 2021 Jun 26;11(7):1904. doi: 10.3390/ani11071904 (PMC8300423; doi:10.3390/ani11071904)
Supplement: Supplementary file 1 [file animals-11-01904-s001.zip › Supplementary tables update.pdf]

Table S1. qPCR performance of the genes analyzed in *Longissimus* muscle.

Gene[1]	Median Ct[2]	Median ΔCt[3]	Slope[4]	(R^2)[5]	Efficiency[6]	Relative mRNA abundance[7]
MYOG	23.845	6.5435	−3.48	0.987	1.937	0.0132
MYOD1	23.943	6.6706	−3.43	0.988	1.958	0.0113
PAX7	29.550	12.2835	−3.25	0.993	2.032	0.0002
MYF5	26.935	9.6754	−3.22	0.995	2.045	0.0010
MYH1	15.355	−2.9116	−3.28	0.992	2.019	7.7358
MYH2	16.995	−1.2911	−3.25	0.999	2.031	2.4961
MYH7	16.173	−1.0751	−3.41	0.997	1.965	2.0676
MEF2C	25.195	7.0340	−3.49	0.994	1.935	0.0096
AGPAT1	26.992	9.7029	−3.19	0.990	2.059	0.0009
PPARGC1A	25.538	8.2646	−3.41	0.990	1.965	0.0038
PPARG	27.927	10.6139	−3.43	0.989	1.958	0.0008
ZFP423	24.021	6.8094	−3.55	0.987	1.913	0.0121
CEBPA	26.395	7.6611	−3.19	0.992	2.056	0.0040
CEBPB	27.030	8.2960	−3.35	0.995	1.989	0.0033
FABP4	21.913	3.4202	−3.19	0.991	2.057	0.0848

[1]*MYOG* Myogenin, *MYOD1* Myogenic differentiation 1, *PAX7* Paired box protein 7, *MYF5* Myogenic factor 5, *MYH1* Myosin heavy chain 1, *MYH2* Myosin heavy chain 2, *MYH7* Myosin heavy chain 7, *MEF2C* Myocyte enhancer factor 2C, *AGPAT1* Acyl-glycerol phosphate acyltransferase 1, *PPARGC1A* PPARG coactivator 1 alpha, *PPARG* Peroxisome proliferator activated receptor gamma, *ZFP423* Zinc finger protein 423, *C/EBPA* CCAAT enhancer binding protein alpha, *C/EBPB* CCAAT enhancer binding protein beta, *FABP4* Fatty acid binding protein 4. [2] The median is calculated considering all steers. [3] The median of ΔCt is calculated as [Ct gene – geometrical mean of Ct internal controls] for each steer. [4] Slope of the standard curve. [5] R^2 stands for the coefficient of determination of the standard curve. [6] Efficiency is calculated as [10(-1 / Slope)]. [7] Relative mRNA abundance = 1/ Efficiency Median ΔCt

Table S2. qPCR performance of the genes analyzed in subcutaneous adipose tissue

Gene [1]	Median Ct [2]	Median ΔCt [3]	Slope [4]	(R^2) [5]	Efficiency [6]	Relative mRNA Abundance [7]
FASN	16.948	−3.2600	−3.37	0.999	1.981	9.2823
SREBP1	22.131	1.9597	−3.22	0.983	2.045	0.2461
PPARG	23.709	3.6456	−3.39	0.998	1.974	0.0839
ADFP	21.908	1.5577	−3.45	0.990	1.948	0.3539
SCD	15.892	−4.5701	−3.55	0.992	1.913	19.3803
FABP4	16.304	−3.8287	−3.40	0.992	1.969	13.3892
PPARGC1A	28.331	−4.5180	−3.30	0.981	2.011	23.4826
ZFP423	23.849	3.2014	−3.55	0.983	1.914	0.1251
ACACA	20.851	0.5347	−3.53	0.995	1.921	0.7053
CEBPA	23.241	3.2014	−3.20	0.996	2.056	0.0995
CEBPB	26.974	6.7941	−3.44	0.990	1.952	0.0106

[1] *FASN* Fatty acid synthase, *SREBP1* Sterol regulatory element binding transcription factor 1, *ADFP* Adipose differentiation-related protein, *SCD* Stearoyl-CoA desaturase, *ACACA* Acetyl-CoA carboxylase alpha. [2] The median is calculated considering all steers. [3] The median of ΔCt is calculated as [Ct gene – geometrical mean of Ct internal controls] for each steer. [4] Slope of the standard curve. [5] R^2 stands for the coefficient of determination of the standard curve. [6] Efficiency is calculated as [10(-1 / Slope)]. [7] Relative mRNA abundance = 1/ Efficiency Median ΔCt
